# Sex- and suicide-specific alterations in the kynurenine pathway in the anterior cingulate cortex in major depression

**DOI:** 10.1038/s41386-023-01736-8

**Published:** 2023-09-21

**Authors:** Samara J. Brown, Katerina Christofides, Christin Weissleder, Xu-Feng Huang, Cynthia Shannon Weickert, Chai K. Lim, Kelly A. Newell

**Affiliations:** 1 School of Medical, Indigenous and Health Sciences and Molecular Horizons, University of Wollongong, Wollongong, NSW Australia; 2 NICM Health Research Institute, Western Sydney University, Westmead, NSW Australia; 3 Schizophrenia Research Laboratory, Neuroscience Research Australia, Randwick, NSW Australia; 4 Mechanism and Therapy of Genetic Brain Diseases, Institut Imagine, Paris, France; 5 Department of Neuroscience & Physiology, Upstate Medical University, Syracuse, NY USA; 6 Discipline of Psychiatry and Mental Health, Faculty of Medicine, University of New South Wales, Sydney, NSW Australia; 7 Macquarie Medical School, Faculty of Medicine, Health and Human Sciences, Macquarie University, Sydney, NSW Australia

**Keywords:** Translational research, Depression, Cellular neuroscience

## Abstract

Major depressive disorder (MDD) is a serious psychiatric disorder that in extreme cases can lead to suicide. Evidence suggests that alterations in the kynurenine pathway (KP) contribute to the pathology of MDD. Activation of the KP leads to the formation of neuroactive metabolites, including kynurenic acid (KYNA) and quinolinic acid (QUIN). To test for changes in the KP, postmortem anterior cingulate cortex (ACC) was obtained from the National Institute of Health NeuroBioBank. Gene expression of KP enzymes and relevant neuroinflammatory markers were investigated via RT-qPCR (Fluidigm) and KP metabolites were measured using liquid chromatography-mass spectrometry in tissue from individuals with MDD (*n* = 44) and matched nonpsychiatric controls (*n* = 36). We report increased *IL6* and *IL1B* mRNA in MDD. Subgroup analysis found that female MDD subjects had significantly decreased KYNA and a trend decrease in the KYNA/QUIN ratio compared to female controls. In addition, MDD subjects that died by suicide had significantly decreased KYNA in comparison to controls and MDD subjects that did not die by suicide, while subjects that did not die by suicide had increased *KYAT2* mRNA, which we hypothesise may protect against a decrease in KYNA. Overall, we found sex- and suicide-specific alterations in the KP in the ACC in MDD. This is the first molecular evidence in the brain of subgroup specific changes in the KP in MDD, which not only suggests that treatments aimed at upregulation of the KYNA arm in the brain may be favourable for female MDD sufferers but also might assist managing suicidal behaviour.

## Introduction

Major depressive disorder (MDD) is a common, complex psychiatric disorder that, in some cases, can lead to suicide. Most suicides worldwide are related to psychiatric disorders, with MDD being one of the most relevant risk factors [1]. The underlying neurobiology of MDD and suicide are unknown but are thought to be outcomes of a complex interaction of molecular changes, environmental stimuli, genetic, and developmental factors. Understanding the complex neurobiology underlying MDD is further complicated as there are sex differences in terms of prevalence and severity [2, 3]. Thus, both sex and manner of death need to be considered in neurobiological studies for MDD.

One of the most consistent findings of MDD is the difference in prevalence rates according to sex, with females on average having double the rates of MDD compared to males [4–6]. In addition, symptom presentation is generally more severe in females [7, 8]. Females with MDD typically experience prolonged or recurrent depression more than males, with a younger onset age and lower quality of life. Notably, therapeutic outcomes also vary between males and females [9, 10]. Although the incidence of sex differences in MDD has been known for over half a century, most molecular studies are not designed to investigate sex-specific changes in MDD. More recently, studies have begun exploring sex differences in MDD, showing that transcriptional abnormalities in cortico-limbic brain regions associated with MDD differ greatly between the sexes [11–14]. It is necessary to build on the limited research aimed at understanding the mechanisms underlying sex-related differences in MDD.

Dysregulation of the kynurenine pathway (KP), involving changes in the concentration of key metabolites and enzymes has been implicated in MDD [15], with evidence of sex differences in non-psychiatric controls [16–18]. The KP is the main catabolic route of tryptophan. Proinflammatory cytokines, including interleukin-1β (IL-1β) and IL-6, activate the first, rate-limiting enzymes of the KP, indoleamine 2,3-dioxygenase 1 (IDO1), IDO2, or tryptophan 2,3 dioxygenase (TDO). Activation of these enzymes, stimulate the metabolism of tryptophan into kynurenine [19]. In the brain, kynurenine is processed by astrocytes or microglia to produce distinct neuroactive compounds, including quinolinic acid (QUIN), kynurenic acid (KYNA), picolinic acid (PIC), and 3-hydroxykynurenine (3-HK) (Fig. 4) [20]. In microglia, kynurenine is metabolised by kynurenine 3-monooxygenase (KMO) into the neuroactive intermediate 3-HK and further metabolised by kynureninase (KYNU) to 3-hydroxyanthanilic acid (3-HAA), which is converted by 3-hydroxyanthranilate 3, 4-dioxygenase (3-HAO) to produce the N-methyl-D-aspartate receptor (NMDAR) agonist, QUIN. In astrocytes, kynurenine is metabolised by kynurenine aminotransferase (KAT) 1-4, producing the NMDAR antagonist, KYNA. Changes in the levels of these neuroactive KP metabolites are likely important in psychiatric symptom generation as both QUIN and KYNA affect glutamatergic neurotransmission, which has been implicated as a potential biological mechanism in MDD [21]. Increased QUIN is considered neurotoxic due to its potential to enhance glutamatergic signalling, whilst increased KYNA is proposed to be neuroprotective by antagonising the NMDAR and reducing glutamatergic signalling. As QUIN and KYNA have opposing effects on the NMDAR, the relative ratio of these metabolites, in addition to their absolute levels, is of particular interest in determining their potential to impact glutamatergic signalling.

Meta-analyses of peripheral KP metabolites have reported that levels of tryptophan, kynurenine and KYNA are decreased in MDD compared to controls [22–25]. Collectively, this suggests a decrease in the activity of the KYNA branch and a subsequent increase in potential activity of QUIN [23, 26]. Meta-regression comparing sex, irrespective of diagnosis, did not identify any specific sex differences in the periphery [23, 24]. However, females with current or lifetime depression have lower levels of tryptophan in both serum and cerebrospinal fluid (CSF) compared to males [17], and the serum kynurenine/tryptophan ratio was shown to predict depressive symptoms in females [27]. Although several studies have examined peripheral levels of the KP metabolites, brain levels have rarely been studied and there is limited investigation of sex-specific alterations of the KP in MDD.

In the brain, studies show increased *KYAT1* and *KYAT2* mRNAs [28], along with increased density of TDO-positive glial cells in the anterior cingulate cortex (ACC) of MDD subjects compared to controls, but no change in tryptophan or kynurenine levels [29, 30]. Furthermore, MDD subjects who died by suicide show increased QUIN-positive cells in the subgenual ACC and anterior-mid cingulate cortex [31]. Evidence has shown that structural and functional damage to the ACC is core to the features of MDD [32, 33]. Abnormal connectivity of the ACC in MDD has also been linked with the peripheral kynurenine pathway [34]. Collectively, these studies highlight the importance of the ACC and provide evidence of an altered KP in MDD and suicide. However, without investigating suicide-specific differences and adequately powering studies to detect sex-specific alterations in the KP in the brain, the understanding of how suicide differs from non-suicide cases and the potential presence of sex-specific changes in the KP remains unclear.

In the present study, we examined postmortem ACC brain tissue from a large cohort of MDD subjects and non-psychiatric controls. Specifically, we measured brain KP metabolites and enzyme gene expression with a specific focus on sex differences. In addition, the activity of the different KP enzymes was assessed by determining various KP metabolite ratios (e.g., KYN/TRP ratio estimating IDO and/or tryptophan 2,3-dioxygenase (TDO) enzyme activity; the KYNA/KYN ratio estimating KAT enzyme activity; the 3-HK/KYN ratio estimating KMO enzyme activity). In parallel, we examined cytokine and glial gene expression, in the same tissues to better understand the relationship between the KP and relevant neuroinflammation markers.

## Methods

### Subject demographics

Postmortem human grey matter from the ACC (Brodmann’s area 24) was obtained from the NIH NeuroBioBank. Specimens were obtained across six biorepositories: University of Miami Brain Endowment Bank, University of Maryland Brain and Tissue Bank, Harvard Brain Tissue Resource Centre, The Human Brain and Spinal Fluid Resource Centre, Mt. Sinai Brain Bank and the Brain Tissue Donation Programme at the University of Pittsburgh. The cohort consisted of 44 individuals with MDD and 36 unaffected comparison subjects (herein referred to as controls). All demographic information and medical data were provided by the NIH NeuroBioBank. This study was approved by the University of Wollongong Human Research Ethics Committee (HE13/069). All groups were matched for the demographic variables, age, postmortem interval (PMI), RNA integrity number (RIN), hemisphere, and sex (all *p* > 0.05) (Table 1). For information on cause of death, see Supplementary Table 1.

**Table 1:** Summary of postmortem subject demographics.

Variable	Control (*n* = 36)			MDD (*n* = 44)			Diagnosis *p*-value	Sex *p*-value	Interaction *p*-value
Sex	F (*n* = 16)	M (*n* = 20)	Combined	F (*n* = 22)	M (*n* = 22)	Combined	0.621	–	–
Age at death (years)	62 ± 16	63 ± 15	62 ± 15	55 ± 18	57 ± 15	56 ± 17	0.096	0.625	0.988
Postmortem interval (hours)	17 ± 7	17 ± 5	17 ± 6	21 ± 10	19 ± 9	20 ± 9	0.116	0.716	0.545
RNA Integrity Number	6 ± 2	6. ± 2	6 ± 2	7 ± 1	6 ± 2	6 ± 2	0.985	0.146	0.545
Suicide (Y/N)	0	0	0	5/14	7/11	12/44	–	0.414	–
Hemisphere (L/R)	7/8	9/10	16/18	15/5	12/8	27/13	0.076	0.433	–

Values are represented as mean ± SD, unless otherwise specified. Data was analysed using a two-way ANOVA including diagnosis and sex as relevant factors. Sex, suicide and hemisphere were analysed using a chi-square test.

*L* left, *R* right, *M* males, *F* females, *Y* Yes, *N* No.

### RNA extraction and qRT-PCR

Total RNA was extracted from samples using TRIzol according to manufacturer guidelines (Invitrogen, Mulgrave, VIC, AUS) (see supplementary methods). RNA was quantified by nanodrop using a ND-1000 Spectrophotometer (Nanodrop Technologies, Wilmington, DE, USA). The RIN was measured for each sample using Agilent Bioanalyzer 2100 (Agilent Technologies, Santa Clara, CA, USA). Complementary DNA was synthesised from 2 μg total RNA using Superscript IV First-Strand Synthesis Kit and random hexamers (Life Technologies, 18091200). TaqMan gene expression assays (Invitrogen) were used to run high-throughput qPCR (Fluidigm; Ramaciotti Centre for Genomics, UNSW, Sydney) to measure KP enzymes, cytokine and glial mRNAs (Supplementary Table 2). Gene expression was quantified using a seven-point standard curve and was normalised to the geometric mean of three housekeeping genes: *GAPDH*, *GUSB* and *TBP*, which did not differ between MDD and controls (t(67) = 0.413, *p* = 0.681) or across the sex*diagnosis groups (F(1,65) = 0.580, *p* = 0.449). The no template control and reverse transcriptase control did not produce a signal in any assay.

### LCMS measurement of metabolites

Tryptophan, kynurenine, 3-HK, 3-HAA, xanthurenic acid (XA), QUIN, KYNA, and formic acid were obtained from Sigma-Merck, while methanol (100% MeOH; Honeywell, LC–MS grade) was sourced from ChemSupply, Australia. Ultra-pure water was obtained from a Milli-Q Direct 9 system (Sigma-Aldrich). The following deuterated internal standards were used: d.AA, d.KYN and d.TRP (CDN Isotopes, Canada).

Fresh frozen brain tissues (~100 mg) were homogenised in a master mix consisting of; 100 µL of ice cold 0.1% formic acid in water, 400 µL ice-cold methanol, and 10 µM of the deuterated internal standards mix. Brain homogenates were stored at −20 °C for 1 h to allow complete protein precipitation. Cellular debris was removed by centrifugation at 12,000 g for 10 min at 4 °C. The tissue lysates (~300 µL) were then dried under vacuum (ThermoFisher, SpeedVac) and resuspended in 100 µL of 0.1% formic acid in water. Detection and quantification of tryptophan, kynurenine, 3-HK, 3-HAA, XA, QUIN, and KYNA were performed using LC-MS (See Supplementary Figs. 1–2). The LCMS-8040 (Shimazdu, Kyoto, Japan) was equipped with a LC-20AD pump, DGU-20A3R degasser, SIL-20A autosampler, and CTO-20AC column oven, coupled with a triple-quadruple mass spectrometer (LCMS-8040) fitted with an ESI interface. 5 μl of each sample was injected into a Luna® PFP(2) 100 Å, (100 × 2 mm, 3 μm) reversed phase analytical column. Samples were eluted at a column temperature of 40 ^o^C and flow rate of 0.5 mL/min, with binary solvents of 0.1% formic acid in water (A) and 100% methanol (B). Positive ion species were detected by mass spectrometry via multiple reaction monitoring (MRM) mode. The mass spectrometry parameters for LCMS-8040 were as follows: nebulizing gas flow at 3 L/min, drying gas flow at 15 L/min, DL temperature at 250°C, heat block temperature at 400 °C, and CID gas at 230 kPa. Overall, the LLOQ was determined based on the linearity and accuracy data. All metabolites were detected at the 20 nM range. Results were normalised to brain tissue mass and expressed as nM/mg tissue weight.

### Antidepressant drug impacts

To examine if chronic antidepressant drug treatment could have impacted gene expression of the key kynurenine pathway enzymes, female Sprague-Dawley rats were treated for 5 weeks with fluoxetine (10 mg/kg) or imipramine (10 mg/kg). Gene expression of *Kyat2* and *Kmo* was analysed using qPCR (for full details see supplementary methods).

### Statistical analysis

SPSS was used for all statistical analysis (Version 28, IBM, Armonk, NY, USA). Extreme outliers were identified in diagnostic and sub-diagnostic groups via boxplot (defined as three times the interquartile range) and excluded. Data for each gene or metabolite was tested for normality using Shapiro–Wilk test and Q-Q plot assessment. When data was not normally distributed, data was natural log transformed to achieve relatively normal distribution. If normal distribution was not achieved, non-parametric analyses were used. To examine sex-diagnosis interactions, in addition to any main diagnosis or sex effects, two-way ANOVAs or two-way ANCOVAs were used. Covariates were identified via Spearman’s correlations between relevant genes/metabolites and PMI, RIN or age in MDD and controls separately. Covariates were subsequently included if they were found to significantly contribute to the two-way ANOVA model. Where a significant interaction was identified, Bonferroni simple main effects were used to determine specific changes. One-way ANOVAs or ANCOVAs with a Bonferroni post-hoc were used to examine suicide subgroup differences (controls, MDD-suicide, and MDD-non-suicide) for gene expression and metabolite levels. Metabolites and genes of interest were investigated for significant correlations using Pearson or Spearman’s correlations where appropriate. Significance was set at *p* < 0.05. All data are presented as means ± SEM.

## Results

### Evidence of sex-specific differences in MDD

**Fig. 1:**
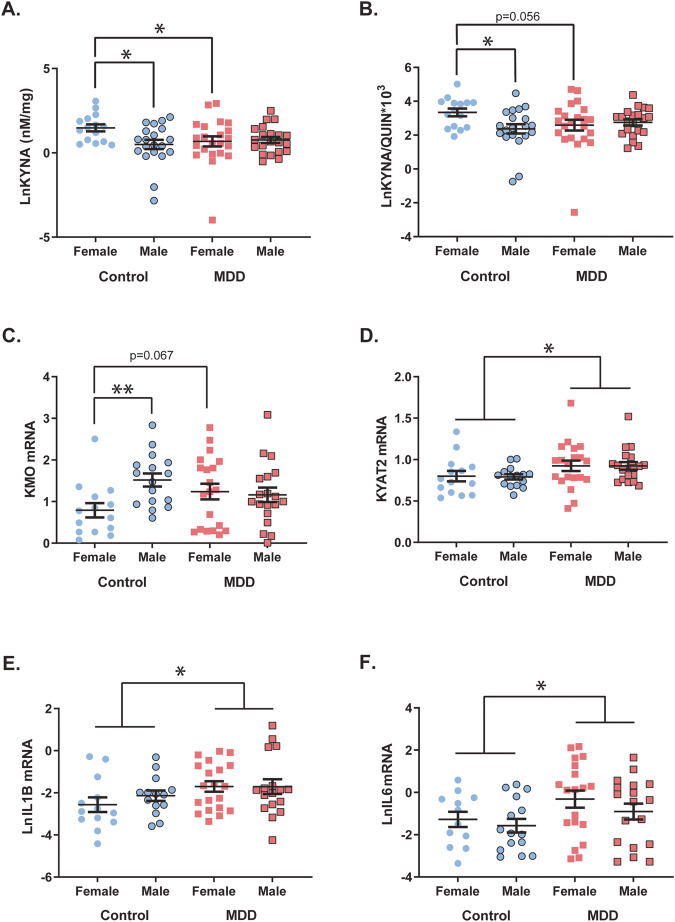
Sex-specific alterations in the kynurenine pathway are present in major depressive disorder. **A** Kynurenic acid (KYNA) was significantly decreased in females with major depressive disorder (MDD) compared to controls (*p* = 0.036). KYNA was significantly higher in female controls compared to male controls (*p* = 0.012). **B** The KYNA/QUIN ratio was significantly higher in female controls compared to male controls (*p* = 0.017). There was a trend decrease in the KYNA/QUIN ratio in females with MDD compared to female controls (*p* = 0.056). **C** *KMO* mRNA was significantly higher in male controls compared to female controls (*p* = 0.004). In females, MDD subjects had a trend increase in *KMO* mRNA compared to female controls (*p* = 0.067). **D** There was a main diagnostic effect for *KYAT2* mRNA. *KYAT2* mRNA was significantly increased in MDD compared to controls (*p* = 0.025). **E** *IL1B* and (**F**) *IL6* mRNAs were significantly increased in MDD compared to controls (*p* = 0.017, *p* = 0.039, respectively). Controls are represented by circles and MDD subjects are represented by squares. Outlined shapes represent male subjects. Bars indicate mean ± SEM. *p < 0.05, **p < 0.01.

There were significant interactions between sex and diagnosis on KYNA (F(1,74) = 4.350, *p* = 0.040) and the KYNA/QUIN ratio (F(1,74) = 4.546, *p* = 0.036) (Fig. 1A, B). Mean KYNA levels were significantly lower in females with MDD (−36.7%) compared to female control subjects (*p* = 0.036). In MDD, females had a trend decrease of the KYNA/QUIN ratio (−35.9%; *p* = 0.056) compared to female controls. In controls, female subjects had higher KYNA (+125.6%) and KYNA/QUIN ratio (+117.5%) levels compared to male controls (*p* = 0.012, *p* = 0.017, respectively).

We identified a significant main effect of sex on 3-HK levels (F(1,72) = 4.141, *p* = 0.046, controlling for PMI) and XA levels (F(1,73) = 4.159, *p* = 0.045), with females having significantly higher levels compared to males for both measures (3-HK: +30.3%; XA: +65.1%) (Supplementary Fig. 3). There was no change in any metabolite levels specifically in males with MDD compared to male controls. In addition, no main effect of sex, or interaction between sex and diagnosis on tryptophan, kynurenine, 3-HAA, QUIN, KYN/TRP, KYNA/KYN or the 3HK/KYN ratio was observed (all *p* > 0.05). There was no main effect of diagnosis on all metabolites measured (*p* > 0.05, see Supplementary Tables 3–4 for full statistics and inclusion of covariates). Sensitivity analyses showed controlling for age and PMI had little effect on results; however, we did see an additional significant effect of sex on kynurenine (Supplementary Table 5 and Supplementary Fig. 4).

There was a significant main effect of sex on *KMO* mRNA (F(1,64) = 5.321, *p* = 0.024, controlling for RIN), and a significant interaction between sex and diagnosis on *KMO* mRNA (F(1,64) = 4.899, *p* = 0.030, controlling for RIN). *KMO* mRNA was significantly higher in male control subjects (+92.1%) compared with female control subjects (*p* = 0.004). In MDD, females showed a trend increase in *KMO* mRNA compared to female controls (*p* = 0.067) (Fig. 1C). There was a main effect of diagnosis on *KYAT2* mRNA (F(1,63) = 5.283, *p* = 0.025, controlling for RIN), with MDD subjects having significantly higher *KYAT2* mRNA (+16.2%) compared to controls (Fig. 1D). No main effect of sex, diagnosis, or interaction between sex and diagnosis on *KYAT1*, *KYNU*, *HAAO* or *QPRT* mRNAs were observed (all *p* > 0.05) (See Supplementary Table 6 for full statistics and inclusion of covariates). In the female Sprague-Dawley rats, treatment with fluoxetine or imipramine did not alter *Kyat2* or *Kmo* mRNAs (Supplementary Fig. 5).

Investigation of the inflammatory cytokines revealed *IL1B* and *IL6* mRNAs were significantly increased in MDD compared to control subjects (*IL1B*: +87.6%, F(1,59) = 6.052, *p* = 0.017, controlling for age; *IL6*: +162.8%, F(1,60) = 4.475, *p* = 0.039) (Fig. 1E, F). There were no main effects of sex or interactions between sex and diagnosis on *IL1B* or *IL6* mRNAs (*p* > 0.05). We identified no interaction between sex and diagnosis on gene expression of the astroglial markers *GFAP* and *AQP4* (F(1,64-65) < 0.233, *p* > 0.631) or microglial markers *AIF1* and *CX3CR1* (F(1,63-65) < 0.885, *p* > 0.350). No main effect of sex or diagnosis were observed for *GFAP*, *AQP4*, *AIF1* or *CX3CR1* mRNAs (all *p* > 0.05). Sensitivity analyses showed controlling for age, PMI and RIN had little effect on results, however, an additional significant effect of diagnosis on *AIF1* was observed (Supplementary Table 7 and Supplementary Fig. 6).

### Suicide-specific changes in the KP

**Fig. 2:**
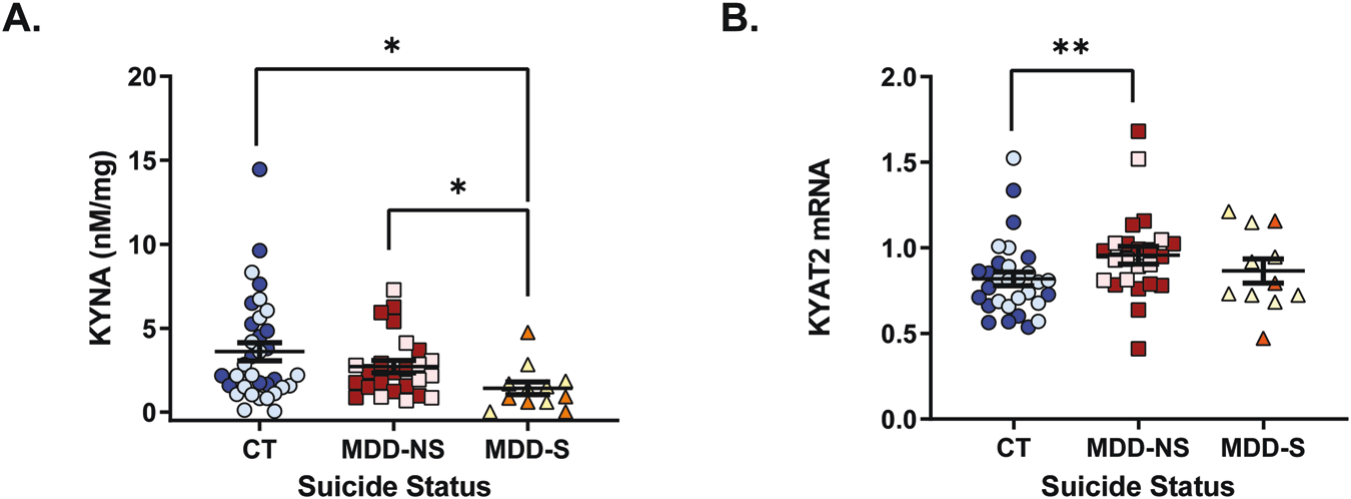
Suicide-specific alterations in the kynurenine pathway. **A** Kynurenic acid (KYNA) was significantly decreased in major depressive disorder subjects that died by suicide (MDD-S) compared to controls (*p* = 0.011) and decreased compared to MDD subjects that did not die by suicide (MDD-NS) (*p* = 0.045). **B** *KYAT2* mRNA was significantly increased in MDD-NS compared to controls (*p* = 0.004). Female subjects are represented by darker colour. Bars indicate mean ± SEM. *p < 0.05, **p < 0.01.

When analysing the cohort by suicide subgroups (control, MDD-suicide, MDD-non-suicide), KYNA levels were significantly different (F(2,68) = 3.434, *p* = 0.038, controlling for PMI), with MDD subjects that died by suicide showing significantly lower (−60.6%) KYNA levels compared to controls (*p* = 0.011) and MDD subjects that did not die by suicide (−48.3%; *p* = 0.045) (Fig. 2A). *KYAT2* mRNA was significantly different across the suicide subgroups (F(2,61) = 4.522, *p* = 0.015, controlling for RIN) (Fig. 2B). MDD subjects that did not die by suicide had elevated *KYAT2* mRNA (+16.8%) compared to controls (*p* = 0.004). No other KP genes or metabolites were significantly different between groups (F(2,48-69) < 1.461, *p* > 0.240). There was a relatively equal spread of males and females across each subgroup and there was no significant difference in the proportion of females across each group (χ^2^ = 1.691, *p* = 0.429, Supplementary Table 8). Sensitivity analyses showed that controlling for age, PMI and RIN had little effect on results with the exception that group differences in KYNA were not significant when both age and PMI were included in the model. However, when either age or PMI was included individually the group difference remained significant (Supplementary Tables 9–10).

### *GFAP* mRNA correlates with *KYAT1* mRNA in the ACC of MDD subjects

To determine how the KP is related to central measures of inflammation in controls and MDD, we ran correlations between metabolites and gene expression. A significant, positive correlation was identified between the astrocyte marker, *GFAP* and *KYAT1* mRNAs in MDD subjects (r_s_ = 0.537, *p* < 0.001, Supplementary Fig. 7B); in controls, we saw a similar patten of correlation, however, this was not significant (r_s_ = 0.314, *p* = 0.091). No significant correlations were found between *GFAP* and *KYAT2* mRNAs in MDD or controls (*p* > 0.05). Furthermore, no significant correlations were found between the microglial marker, *AIF1 (IBA1*) and *KMO* mRNAs in either MDD or controls (all *p* > 0.05). In controls, *IL6* mRNA was positively correlated with KYNA (r_s_ = 0.576, *p* = 0.001) and kynurenine (r_s_ = 0.757, *p* < 0.001). In MDD *IL1B* mRNA was positively correlated with kynurenine (r_s_ = 0.382, *p* = 0.018). For metabolite-metabolite correlations and correlations between representative enzyme activity ratios (metabolite ratios) and relevant gene expression data, see Supplementary Tables 11–12.

### QUIN positively correlates with age in MDD

**Fig. 3:**
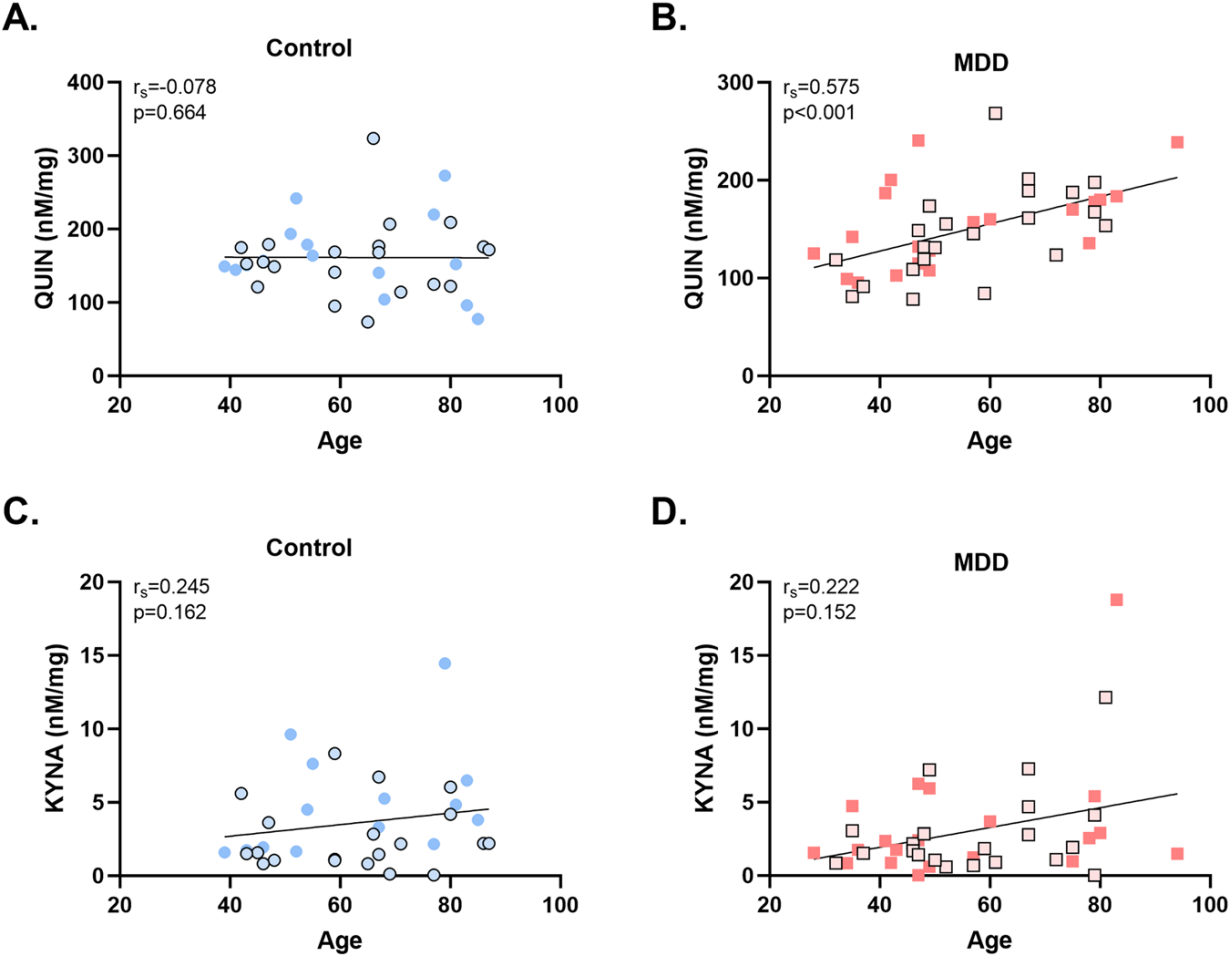
Quinolinic acid is positively correlated with age in major depressive disorder. **A** There was no significant correlation between quinolinic acid (QUIN) and age in controls (*p* = 0.664). **B** In major depressive disorder (MDD) there was a strong positive correlation between QUIN and age (r_s_ = 0.575 *p* < 0.001). **C** There was no correlation between age and kynurenic acid (KYNA) in controls or (**D**) in MDD. Males are represented by the outlined shapes.

To explore the impact of ageing on the metabolites and gene expression, we ran Spearman’s correlations in controls and MDD subjects separately (See Supplementary Table 13). There was a significant positive correlation between QUIN and age in MDD subjects (r_s_ = 0.575, *p* < 0.001) but not in controls (r_s_ = −0.078, *p* = 0.664) nor between KYNA and age (*p* > 0.05) (Fig. 3). There was a significant positive correlation between *KYAT1* mRNA and age in both MDD (r_s_ = 0.452, *p* = 0.012) and controls (r_s_ = 0.424, *p* = 0.008). Furthermore, there was a significant positive correlation between *GFAP* mRNA and age in both MDD (r_s_ = 0.542, *p* = 0.002) and controls (r_s_ = 0.525, *p* < 0.001) (Supplementary Fig. 8).

## Discussion

**Fig. 4:**
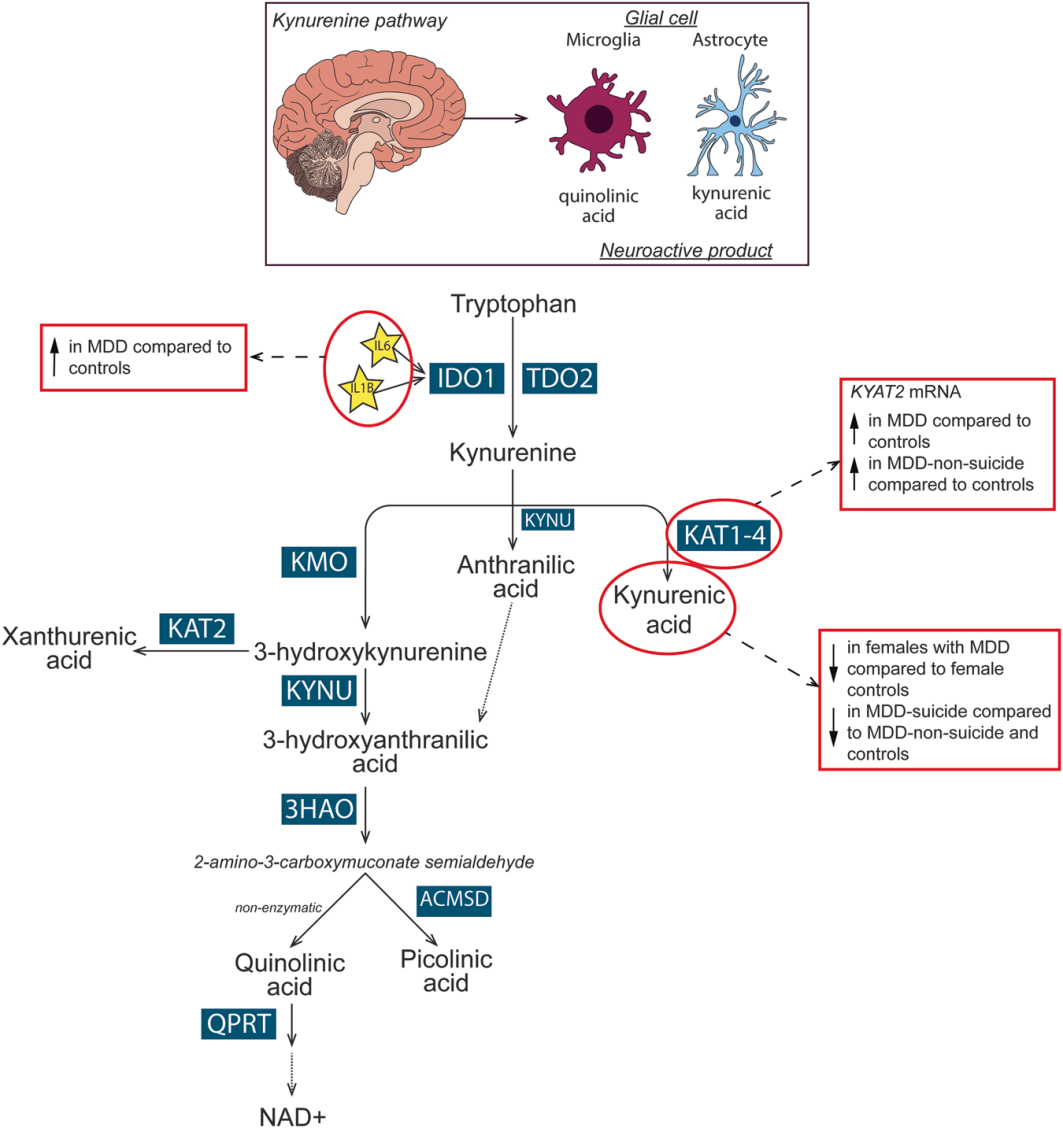
Overview of the kynurenine pathway in the brain and key findings in MDD. In the brain, tryptophan can be metabolised in glial cells via the kynurenine pathway. Dependent on the cell type different neuroactive metabolites will be produced. Predominantly in microglia, kynurenine is metabolised into quinolinic acid whereas kynurenic acid is primarily produced in astrocytes. In the anterior cingulate cortex, *IL6*, *IL1B* and *KYAT2* mRNAs were increased in major depressive disorder (MDD) overall. *KYAT2* mRNA was increased in MDD subjects that did not die by suicide in comparison to controls. Kynurenic acid was decreased in females with MDD in comparison to female controls and was decreased in MDD subjects that died by suicide in comparison to MDD-non-suicide and controls. Abbreviations: 3-HAO 3-hydroxyanthranilate 3,4-dioxygenase, ACMSD α-amino-β-carboxymuconate-ε-semialdehyde, IDO1 indoleamine 2, 3-dioxygenase, IL interleukin, KAT kynurenine aminotransferase, KMO kynurenine 3-monoxygenase, KYNU kynurinase, MDD major depressive disorder, NAD+ nicotinamide adenine dinucleotide, TDO tryptophan 2,3-dioxygenase, QPRT quinolinic acid phosphoribosyltransferase.

This is the first study to comprehensively examine both arms of the KP in MDD, with a specific focus on subgroups, in a large postmortem human brain cohort. In the ACC, female MDD subjects had significantly decreased KYNA and a trend decrease in the KYNA/QUIN ratio compared to female controls. In addition, we report MDD subjects that died by suicide had significantly decreased KYNA levels in comparison to controls and MDD subjects that did not die by suicide. Furthermore, there was a significant increase in *KYAT2* mRNA in MDD, specifically in those that did not die by suicide. In MDD overall, there was a significant increase in *IL6* and *IL1B* mRNAs in the ACC. Collectively, our findings suggest the KP is implicated in female MDD subjects and MDD-suicide in the ACC (Fig. 4).

### Sex-specific changes in MDD and controls

Our data shows evidence of sex-specific changes to the KP in MDD. Specifically, we found that females with MDD have significantly lower KYNA levels compared to female controls. Furthermore, we identified a trend decrease in the KYNA/QUIN ratio in female MDD subjects. This data may indicate a dysfunction of the KP in the ACC that is specific to females with MDD, whereby there is a significant decrease in KP activity. The decreased KYNA and the KYNA/QUIN ratio in female MDD subjects could indicate greater potential of QUIN to modulate glutamatergic signalling towards a hyperglutamatergic environment in the ACC. Furthermore, both KYNA and the KYNA/QUIN ratio are associated with distinct connectivity patterns related to the default mode network [34]. Thus, our findings could suggest impacted functional connectivity may be present in females with MDD. Decreased plasma KYNA has previously been identified as a diagnostic predictor of depression [35, 36] and meta-analyses report that peripheral KYNA levels and the KYNA/QUIN ratio are decreased in MDD [23–25]. Additionally, the serum KYNA/QUIN ratio is negatively associated with symptom severity [37, 38]. Importantly, this evidence is predominantly from peripheral measures and whilst sex is commonly used as a covariate, direct investigation of the interaction between sex and diagnosis is largely lacking. However, one study identified that a decrease in the KYNA/QUIN ratio in MDD was driven by changes specifically in the male cohort [37], in contrast to our present findings.

In addition to the female-specific changes in MDD, we report significant sex differences in controls. We identified that female controls had significantly higher KYNA and KYNA/QUIN ratio compared to male controls. Furthermore, females in this study overall (combined MDD and controls) had higher levels of XA and 3-HK, accompanied by higher gene expression of *KMO* in control males compared to control females. This finding was interesting as we would expect a subsequent increase of 3-HK in males rather than in females. However, this could indicate altered activity levels of the KMO enzyme or possible rapid degradation of the enzyme, contributing to the decreased metabolite levels in males. In addition, the opposite pattern of results between *KMO* mRNA and 3-HK levels between males and females is interesting and potentially highlights a discordance between mRNA and protein expression of the KP enzymes in this study. Collectively, our findings suggest under physiological conditions, that females show greater metabolism of kynurenine in the ACC into both branches of the KP compared to males. Considering women in general have a higher incidence of depression, these changes may be of importance with some parts of the development of MDD [17]. In contrast with our brain findings, plasma and serum kynurenine and KYNA are significantly decreased in healthy females compared to males [16–18, 39]. The discordance between results could be due to the inability of KYNA to cross the blood brain barrier [40].

### Suicide-specific changes in the KP

Previously, dysregulation of the KP has been linked to suicidal behaviour and pathology [31, 41, 42]. Therefore, we explored neurobiological changes specific to MDD-suicide. We identified that those that died by suicide had significantly decreased KYNA in comparison to both controls and MDD subjects that died by other causes. Consistent with our findings in the brain, decreased KYNA has also been reported in the CSF of those that attempted suicide, and low levels correlated with more severe depressive and suicidal symptoms [43]. The observed decrease in KYNA specific to suicide and females with MDD in this cohort is interesting given we identified significant increases in *KYAT2* mRNA in MDD overall, which may suggest the opposite i.e., increased KYNA. Further subgroup analysis identified the increased *KYAT2* mRNA was only significant in MDD subjects that did not die by suicide. Whilst this may be related to a power issue with the small sample size in our MDD suicide cohort, we have previously shown in an independent MDD cohort, that *KYAT2* mRNA was increased to a greater degree in MDD-non-suicide [28]. Similarly, to 3-HK and *KMO* mRNA we see an opposite pattern of results for *KYAT2* mRNA and KYNA. We hypothesised that an increase in *KYAT2* mRNA would result in increased KYNA, as is observed in schizophrenia [44]. However, we did not detect increased KYNA in the current study. KAT 2, the corresponding enzyme of *KYAT2* mRNA, is the major enzyme responsible for KYNA production. Collectively, these results suggest that KAT2 could be dysfunctional and/or working at a reduced rate in MDD or that KYNA is not stable in MDD. One possible explanation for putative blunted KAT2 enzyme activity may be due to alterations in the binding sites resulting in less efficient conversion of kynurenine into KYNA. This may explain why we see a decrease in KYNA specifically in MDD-suicide as these subjects do not have the increased *KYAT2* mRNA (hypothesised to lead to increased enzyme levels) to compensate for a possible reduced efficiency. Furthermore, the discordance in results may be related to the intricate interplay of KP signalling, where additional stimuli could modulate the activity and synthesis of KAT2. Further analysis of enzyme activity or protein expression is warranted to interrogate potential alterations at the protein activity level in MDD, particularly in subjects that died by suicide.

### Increased cytokine gene expression in MDD is not associated with increased downstream KP metabolites

In this study, we found increased gene expression of *IL6* and *IL1B,* which can activate the rate-limiting enzymes of the KP, in MDD. This finding was surprising, as we would have expected to see subsequent increases in kynurenine or some of its downstream metabolites. In contrast to our results, reduced KP activity was associated with decreased cytokine expression in the VLPFC [45]. Our data suggests a disconnect in the relationship between KP activity and cytokines in the ACC in MDD. Furthermore, increased cytokines can shift KP metabolism down the QUIN arm, via activation of KMO [46]. This would explain our decrease in KYNA; however, not our lack of change/increase to QUIN. Despite showing no change in QUIN levels in the ACC in MDD, our findings support the hypothesis of greater QUIN potential in the ACC, as decreased KYNA may favour greater QUIN activity at the NMDAR. Increased QUIN-positive microglial cells have previously been reported in the ACC but decreased in the hippocampus in postmortem brains of MDD subjects who died by suicide [31, 47]. Our null QUIN findings could be due to the short half-life of QUIN as it gets rapidly broken down by the QPRT enzyme [20] or QUIN levels in the ACC may be stable but be spread across different cellular compartments (i.e., microglia, synaptic space, lysosomes) as we measured KP metabolites in homogenous brain samples. While we saw no changes in QUIN levels per se in depression, we saw a positive correlation between age and QUIN only in MDD. This could suggest that the KP is differentially regulated in ageing between the diagnoses, where we see an increase in QUIN in ageing in MDD, which could be related to the progression of depression in later life. As expected, *GFAP* mRNA was positively correlated with age in both MDD and controls [48]. Furthermore, *KYAT1* mRNA was also positively correlated with age, which may suggest increased *KYAT1* mRNA may go hand in hand with increased astrocyte reactivity in the ageing human cerebral cortex.

## Limitations

One limitation of this study is that we did not have sufficient information on antidepressant medication to consider the impact of treatment. However, when we examined chronic antidepressant treatment in female Sprague-Dawley rats, *Kyat2* and *Kmo* mRNAs were unchanged. Furthermore, preclinical evidence shows that antidepressant drugs reduce QUIN and increase KYNA [49, 50]. As our findings showed decreased KYNA in MDD subgroups, it is unlikely that these findings are the result of premortem antidepressant treatment. Furthermore, whether these findings are generalisable to other brain regions requires further examination. BA24 was used for this study, however, a subset of control (*n* = 8) and MDD (*n* = 8) cases also contained the neighbouring ACC region, BA32. Other limitations include our current inability to rule out the possibility that comorbid diagnoses or cause of death may be impacting our measures due to a lack of statistical power. However, our diagnostic groups were matched for cause of death. In addition, oral contraceptive use, exercise, and diet have been shown to impact the KP peripherally [51–53]. Therefore, as with all postmortem human brain studies, there is potential for these factors to influence our results. Lastly, our investigation of the enzymes of the KP was limited to gene expression and it is unclear if these changes would be reflected at the protein level.

## Conclusions

This is the first molecular evidence in the brain of subgroup specific alterations in the KP in the brain in MDD. In the ACC, we identified that KYNA and the KYNA/QUIN ratio were decreased in female MDD subjects, and that KYNA was decreased in those that died by suicide, collectively suggesting that KP activity in the ACC is reduced. This was surprising given we saw an increase in *KYAT2* mRNA and cytokine mRNAs in the same tissue. These opposing results suggest discordance between mRNA and protein levels may exist, or that additional factors may be contributing to the metabolite levels of the KP. Our subgroup findings not only suggest that treatments aimed at upregulation of the KYNA arm in the brain may be favourable for female MDD sufferers but also might assist managing suicidal behaviour. Drugs that increase cerebral KYNA and show rapid antidepressant effects in preclinical models [54, 55] have recently moved to clinical trials but did not show favourable outcomes [56]. However, future trials need to consider the importance of subgroups when selecting the most suitable cohorts. Together our findings show increased inflammation is present in the ACC in MDD coupled with sex- and suicide-specific alterations in the KP. These findings could inform future novel treatment approaches in MDD.

### Supplementary information

Supplementary methods and results
