# Supplementary material for: Sex- and suicide-specific alterations in the kynurenine pathway in the anterior cingulate cortex in major depression
Source: Neuropsychopharmacology. 2023 Sep 21;49(3):584–92. doi: 10.1038/s41386-023-01736-8 (PMC10789861; doi:10.1038/s41386-023-01736-8)
Supplement: Supplementary file 1 — Supplementary methods and results [file 41386_2023_1736_MOESM1_ESM.pdf]

Supplementary Methods

Supplementary Table 1: Summary of cause of death.

Cause of Death:	Control (n=36)	MDD (n=44)	χ^2=7.465	p=0.280
Accidental	1	2		
Cardiac arrest	7	6		
Other cardiac	10	6		
Suicide	0	12		
Cancer	5	1		
Natural	1	2		
Other	4	10		
Unknown	8	5		

Note: Chi-square analysis excluded suicide as a cause of death.

Human ACC RNA Extraction:

Tissue was homogenised in 800µl of cold TRIzol using a disposable tissue pestle (Axygen, Mount Martha, VIC, Australia) and incubated at room temperature for 5 minutes. 160µL of chloroform was added to the sample and then centrifuged at 12,000g for 15 minutes at 4°C. The aqueous phase was transferred to a new tube and 400µl of isopropanol was added, incubated for 10 minutes, followed by centrifugation at 12,000g for 10 minutes at 4°C. After removing the isopropanol, the remaining pellet was washed in 800µL of 70% ethanol and then centrifuged at 7500g for 5 minutes at 4°C. Ethanol was removed and samples were air-dried. The RNA was resuspended in 30µl RNAse-free water (Sigma-Aldrich, Castle Hill, NSW, AUS).

Supplementary Table 2: TaqMan gene expression codes

Gene	Alias	Corresponding protein	Accession number
AIF1		Allograft inflammatory factor 1	Hs_00741549_g1
AQP4		Aquaporin 4	Hs_00242342_m1
CX3CR1		C-X3-C motif chemokine receptor 1	Hs_01598433_m1
GAPDH		Glyceraldehyde-3-phosphate dehydrogenase	Hs_99999905_m1
GFAP		Glial fibrillary acidic protein	Hs_00909236_m1
GUSB		Glucuronidase beta	Hs_99999908_m1
HAAO	3-HAO	3-hydroxyanthranilate 3,4-dioxygenase	Hs_00201915_m1
IL6		Interleukin 6	Hs_00174131_m1
IL1B		Interleukin 1 beta	Hs_01555410_m1
KMO		Kynurenine 3-monooxygenase	Hs_00175738_m1
KYAT1		Kynurenine aminotransferase 1	Hs_00187858_m1
KYAT2	AADAT	Kynurenine aminotransferase 2	Hs_00212039_m1
KYNU		Kynurinase	Hs_011114099_m1
QPRT		Quinolinate phosphoribosyltransferase	Hs_00204757_m1
TBP		TATA-box binding protein	Hs_00427620_m1

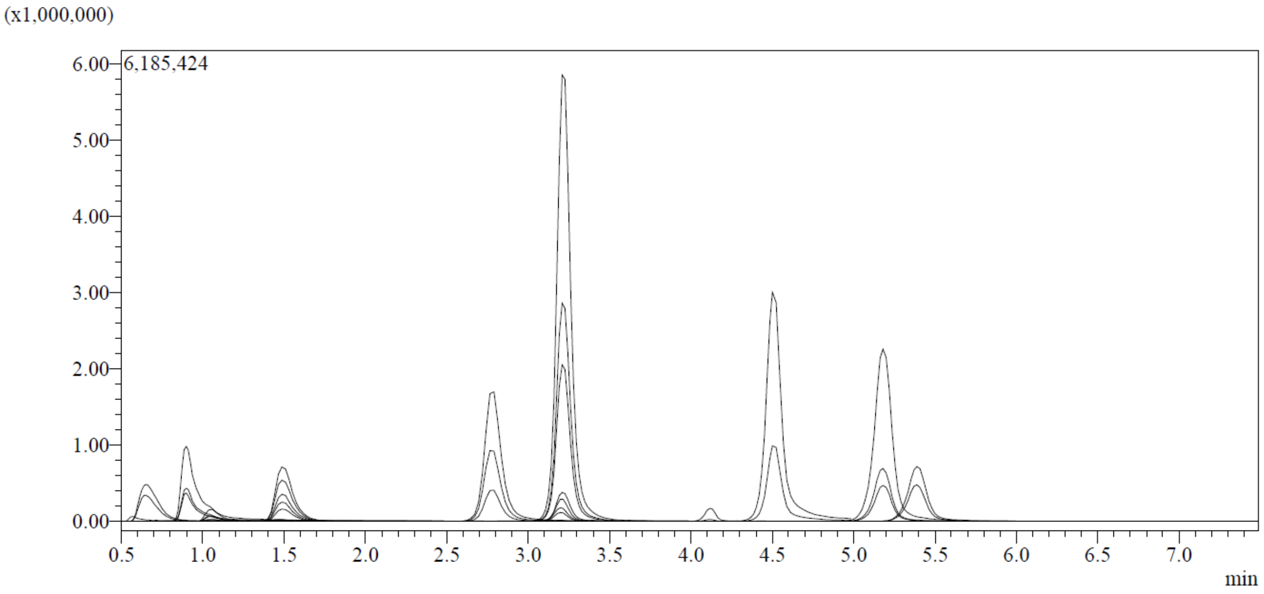

Supplementary Figure 1: Combined Total Ion Chromatogram (TIC) with all metabolites inclusive of their respective optimised MRMs at 10uM mixed Standard. X-axis represents time (sec), Y-axis represents raw intensity. Order of metabolites and their RT (sec): CRE 0.64, 3-HK 1.05, QUIN 0.56, PIC 0.88, KYN 1.49, 5HIAA 1.49, dKYN 1.48, 3HAA 2.77, TRP 3.21, dTRP 3.20, AA 4.50, dAA, 4.43, KYNA 5.18, XA 5.38.

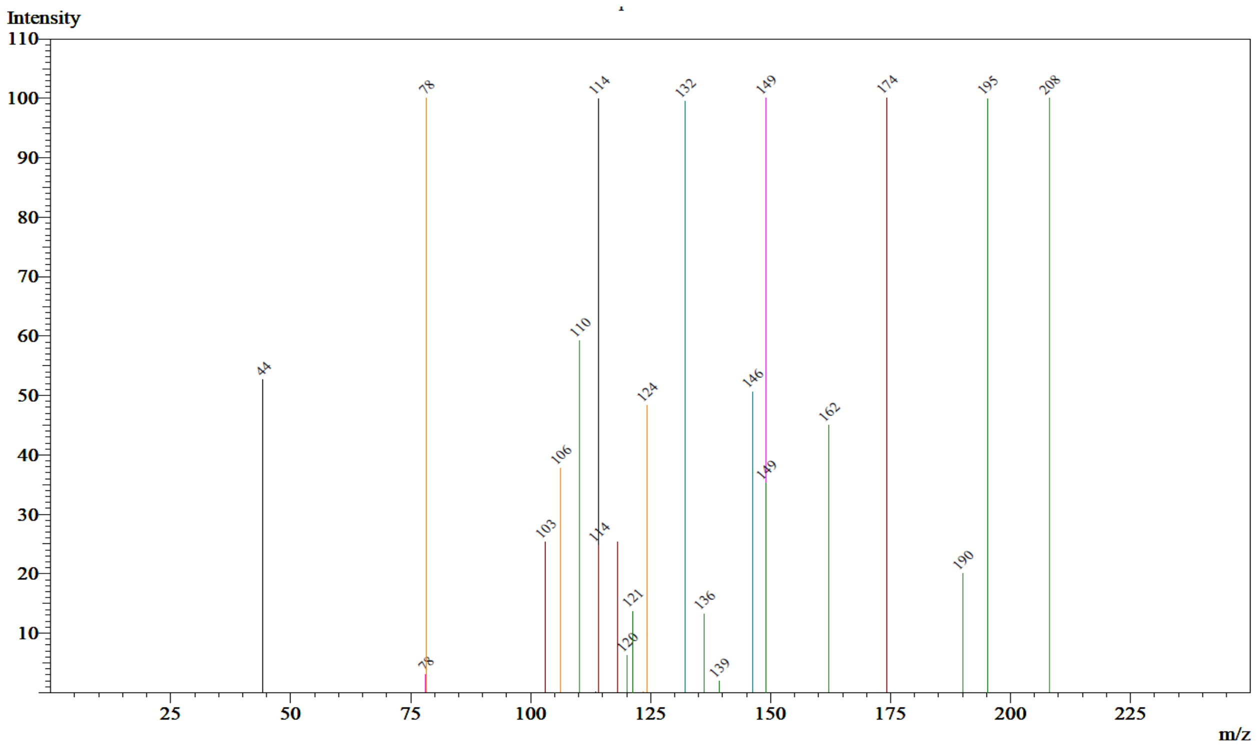

Supplementary Figure 2: Mass spectrum of all metabolites (including MRMs) from the combined method using 10uM mixed Standard. Relative abundance vs detected mass. Order of metabolites and their detected TIC mass (g/mol): CRE 113.60, 3-HK 224.70, QUIN 167.00, PIC 123.60, KYN 208.70, 5HIAA 191.70, dKYN 212.75, 3HAA 153.65, TRP 204.90, dTRP 207.75, AA 137.65, dAA, 141.65, KYNA 189.65, XA 205.65.

<u>Impact of chronic antidepressant treatment in female Sprague-Dawley rats</u>:

Adult female Sprague-Dawley rats (12 weeks of age) were obtained from the Animal Resource Centre (Perth, Australia) and allowed to acclimate for one week. Food and water were provided ad libitum. Rats were randomly assigned to a treatment group (n=10/group): fluoxetine (10mg/kg), imipramine (10mg/kg) or vehicle (saline injection 0.9% NaCl). Treatments were administered once daily via intraperitoneal injections between 0800 and 0900 for 5 weeks. Rats were euthanized via carbon dioxide asphyxiation 24 hours after the last treatment to allow a drug washout period. The brains were immediately removed, snap frozen in liquid nitrogen, and stored at -80°C. Approval for animal experimental procedures was granted by UOW Animal Ethics (ethics number: AE14/01).

Total RNA was isolated from approximately 20 mg of frozen frontal cortex tissue using Purelink RNA Mini Kits (ThermoFisher, 12183025). Quality of the extraction was measured via Nanodrop. Complementary DNA was synthesized from 2µg total RNA using Superscript IV First-Strand Synthesis Kit and random hexamers (Life Technologies, 18091200). The mRNA expression of *Kmo* and *Kyat2*, in the rat frontal cortex was measured by reverse transcriptase–qPCR (Quant Studio 5). Quantitative real-time PCR was performed in a 20-µL final reaction volume using SensiFAST SYBR No-ROX Kit (Bioline, BIO-98020). Amplification was carried out with 40 cycles of 95 °C for 5 seconds, 60 °C for 10 seconds, and 72 °C for 10 seconds. Transcript quantities were normalized to the geometric mean of three housekeeper genes: *Ubc, Gusb* and *B2m*, which did not differ across the treatment groups. The 'no template control' and reverse transcriptase control did not produce a signal in any assay. All amplifications from each subject were performed in triplicate and relative quantities of expression for each gene was calculated using the comparative threshold cycle value (Ct) method, using the formula 2−ΔΔCt (where ΔΔCt = ΔCt sample–ΔCt reference).

Supplementary Results

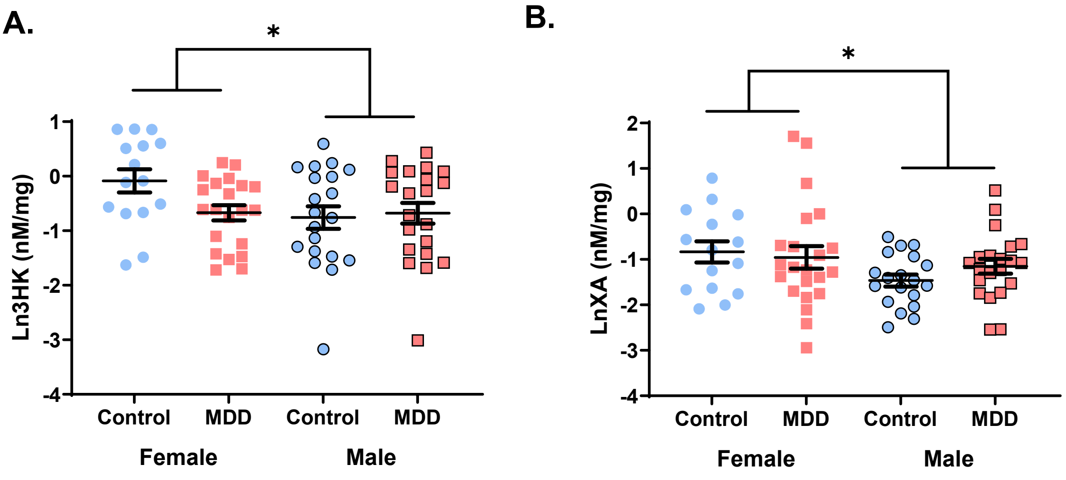

Supplementary Figure 3: (**A**) 3-hydroxykynurenine (3-HK) was significantly lower in males overall compared to females (p=0.046). (**B**) Xanthurenic acid (XA) was significantly lower in males overall compared to females (p=0.045). Ln=natural log; MDD=major depressive disorder. *p<0.05.

Supplementary Table 3: Sex-diagnosis interaction group means for kynurenine metabolites.

Metabolites	Control						MDD					
	Female			Male			Female			Male		
	n	Mean (nM/mg)	SD	n	Mean (nM/mg)	SD	n	Mean (nM/mg)	SD	n	Mean (nM/mg)	SD
TRP	15	384.42	177.38	20	301.43	128.44	22	313.88	132.86	22	313.21	144.78
LnKYN	15	1.71	0.91	20	0.87	1.27	21	1.03	0.92	21	1.02	1.14
LnKYNA	15	1.48	0.81	20	0.49	1.23	22	0.68	1.39	21	0.76	0.81
LnQUIN	15	5.04	0.34	20	5.03	0.31	22	5.00	0.27	22	4.94	0.32
Ln3HK	15	-0.09	0.82	20	-0.76	0.92	21	-0.67	0.64	21	-0.68	0.88
Ln3HAA	14	-2.14	0.89	19	-2.62	0.77	18	-2.57	0.63	18	-2.61	0.71
LnXA	15	-0.83	0.90	19	-1.46	0.57	22	-0.96	1.16	21	-1.15	0.74
LnKYN/TRP x10^3	15	2.81	0.85	20	2.27	0.90	21	2.22	0.93	22	2.28	0.91
LnKYNA/QUIN x10^3	15	3.34	0.88	20	2.37	1.24	22	2.59	1.48	21	2.75	0.83
LnKYNA/KYN	15	6.67	0.39	20	6.53	0.39	21	6.78	0.47	21	6.54	0.36
Ln3HK/KYN	15	5.12	0.79	20	5.28	1.10	21	5.21	0.87	21	5.26	0.88
LnQUIN/KYN	15	3.33	0.97	20	4.16	1.30	21	3.95	0.89	21	3.90	1.12

Supplementary Table 4: Main diagnosis output for kynurenine metabolites from sex*diagnosis two-way ANCOVAs.

Metabolites	Covariate	Comparison
TRP		F(1,75)=0.797, p=0.375
LnKYN	PMI	F(1,72)=0.428, p=0.515
LnKYNA		F(1,74)=1.065, p=0.306
LnQUIN	Age	F(1,74)=0.481, p=0.490
Ln3HK	PMI	F(1,72)=0.783, p=0.379
Ln3HAA		F(1,65)=1.296, p=0.259
LnXA		F(1,73)=0.221, p=0.639
LnKYN/TRP x10^3		F(1,74)=1.974, p=0.164
LnKYNA/QUIN x10^3		F(1,74)=0.503, p=0.481
LnKYNA/KYN		F(1,73)=0.375, p=0.542
Ln3HK/KYN		F(1,73)=0.035, p=0.851
LnQUIN/KYN		F(1,73)=0.512, p=0.477

Supplementary Table 5: P-values for factors included in the linear mixed effects models for the metabolite levels across sex and diagnosis with age and PMI included as covariates.

Measures	Age	PMI	Sex	Diagnosis	Sex by Diagnosis
LnKYNA	0.239	0.156	0.060	0.603	0.049
LnKYNA/QUIN	0.552	0.337	0.117	0.693	0.044
3-HK	0.450	0.005	0.050	0.325	0.117
XA	0.104	0.514	0.046	0.810	0.314
TRP	0.635	0.574	0.232	0.297	0.206
LnKYN	0.070	0.028	0.048	0.741	0.113
Ln3HAA	0.248	0.869	0.151	0.362	0.240
LnKYN/TRP	0.004	<0.001	0.101	0.728	0.158
LnKYNA/KYN	0.037	0.414	0.061	0.935	0.647
Ln3-HK/KYN	0.075	0.603	0.497	0.818	0.804
QUIN	0.026	0.167	0.458	0.786	0.631

Abbreviations: KYNA: kynurenic acid; KYNA/QUIN: ratio of kynurenic acid to quinolinic acid; 3-HK: 3-hydroxykynurenine; XA: xanuthernic acid; TRP: tryptophan; KYN: kynurenine; QUIN: quinolinic acid.

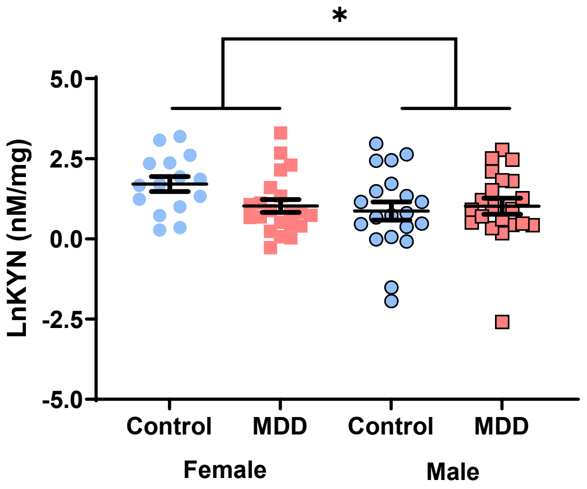

Supplementary Figure 4: After controlling for age and PMI, a significant main effect of sex was observed for kynurenine (p=0.048). *p<0.05.

Supplementary Table 6: Main diagnosis output for kynurenine enzyme mRNA from sex*diagnosis two-way ANCOVAs.

**mRNA**	**Covariate**	**Comparison**
KYAT2	RIN	F(1,63)=5.283, p=0.025
LnKYAT1	Age/PMI	F(1,62)=0.008, p=0.928
KYAT3	Age/RIN	F(1,63)=0.046, p=0.831
KYAT4	RIN	F(1,64)=1.729, p=0.193
KMO	RIN	F(1,64)=0.206, p=0.652
LnKYNU	Age	F(1,62)=3.167, p=0.08
LnHAAO	Age/RIN	F(1,63)=0.020, p=0.887
LnQPRT	Age/RIN	F(1,63)=0.265, p=0.609

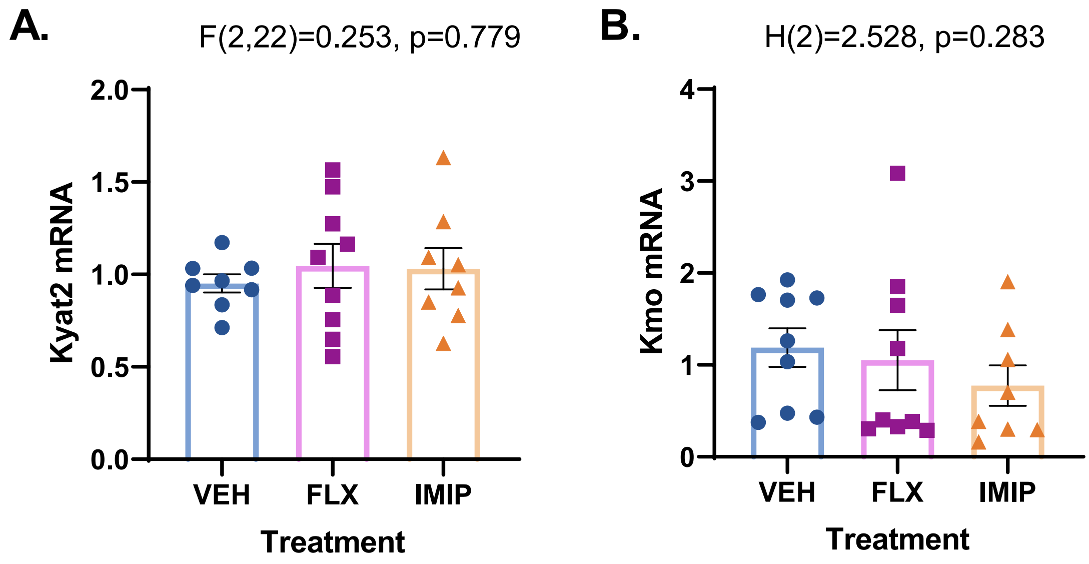

Supplementary Figure 5: There were no changes in (**A**) *Kyat2* or (**B**) *Kmo* mRNAs in the frontal cortex after 5 weeks of treatment with fluoxetine (FLX) or Imipramine (IMIP) in female Sprague-Dawley rats.

Supplementary Table 7: P-values for factors included in the linear mixed effects models for the gene expression across sex and diagnosis with age, PMI and RIN included as covariates.

Measures	age	PMI	RIN	Sex	Diagnosis	Sex by Diagnosis
KYAT2	0.262	0.047	<0.001	0.448	0.016	0.835
KMO	0.042	0.824	0.020	0.019	0.911	0.027
lnIL6	0.108	0.201	0.637	0.206	0.010	0.711
LnIL1B	0.027	0.430	0.117	0.501	0.007	0.693
LnKYAT1	<0.001	0.009	0.132	0.103	0.973	0.880
LnKYNU	0.003	0.214	0.604	0.646	0.051	0.752
LnHAAO	0.669	0.317	<0.001	0.054	0.882	0.669
LnQPRT	0.002	0.893	0.087	0.092	0.539	0.147
LnAIF1	0.011	0.010	0.111	0.128	0.049	0.693
LnGFAP	<0.001	0.615	0.002	0.567	0.673	0.707
LnAQP4	0.072	0.023	0.152	0.905	0.690	0.556
LnCX3CR1	0.313	0.089	0.474	0.109	0.492	0.330

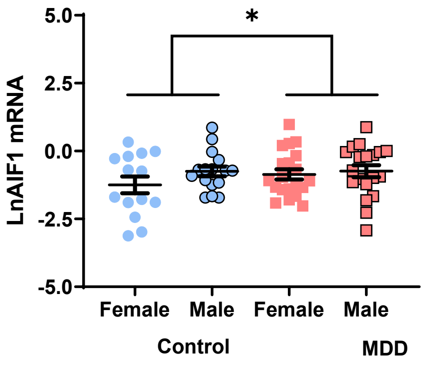

Supplementary Figure 6: After controlling for age, PMI and RIN, a significant main effect of diagnosis was observed for *AIF1* mRNA (p=0.049). *p<0.05.

Supplementary Table 8: Summary of postmortem subject demographics in suicide subgroups.

Variable	Control (n=36)	MDD-NS (n=27)	MDD-S (n=11)	Test statistic	p-value
**Age at death (years)**	62.67±2.51	57.63±17.87	49.18±12.87	H=5.631	0.052
**Postmortem interval (hours)**	17.03±0.99	17.55±7.47	23.59±9.82	H=5.838	0.045
**RNA Integrity Number**	6.15±0.26	6.11±1.71	6.56±1.03	F=0.371	0.691
**Sex**	20M, 16F	11M, 16F	7M, 5F	χ^2=1.691	0.429

Values are represented as mean ± SD, unless otherwise specified. L: left; R: right; M: males; F: females

Supplementary Table 9: P-values for factors included in the linear mixed effects models for the metabolite levels across suicide subgroups with age and PMI included as covariates

**Measures**	**Age**	**PMI**	**Suicide**
KYNA	0.353	0.229	0.105
KYNA/QUIN	0.688	0.451	0.412
3-HK	0.709	0.051	0.157
XA	0.143	0.928	0.547
TRP	0.299	0.558	0.297
KYN	0.452	0.091	0.623
Ln3HAA	0.299	0.948	0.506
LnKYN/TRP	0.015	0.005	0.673
LnKYNA/KYN	0.034	0.276	0.355
Ln3-HK/KYN	0.032	0.684	0.518
QUIN	0.027	0.198	0.921

Abbreviations: KYNA: kynurenic acid; KYNA/QUIN: ratio of kynurenic acid to quinolinic acid; 3-HK: 3-hydroxykynurenine; XA: xanthurenic acid; TRP: tryptophan; KYN: kynurenine; QUIN: quinolinic acid.

Supplementary Table 10: P-values for factors included in the linear mixed effects models for the gene expression across suicide subgroups with age, PMI and RIN included as covariates.

Measures	Age	PMI	RIN	Suicide
KYAT2	0.560	0.148	<0.001	0.021
KMO	0.133	0.814	0.037	0.970
LnIL6	0.769	0.160	0.563	0.191
LnIL1B	0.133	0.297	0.740	0.061
KYAT1	<0.001	0.030	0.134	0.931
LnKYNU	0.008	0.144	0.699	0.212
LnHAAO	0.702	0.817	<0.001	0.945
LnQPRT	0.012	0.855	0.101	0.634
LnAIF1	0.027	0.089	0.165	0.191
LnGFAP	<0.001	0.447	<0.001	0.331
LnAQP4	0.069	0.029	0.108	0.762
LnCX3CR1	0.350	0.311	0.668	0.863

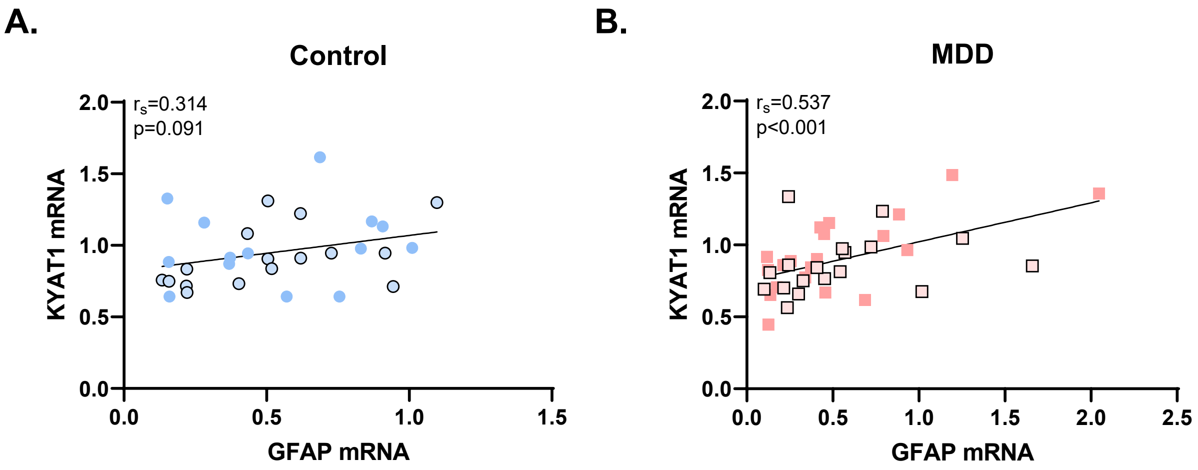

Supplementary Figure 7: (**A**) There was no significant correlation between *KYAT1* and *GFAP* mRNAs in controls (p=0.091). (**B**) In major depressive disorder (MDD) there was a strong positive correlation between *KYAT1* and *GFAP* mRNAs (r_s=0.537 p<0.001). Males are represented by the outlined shapes.

Supplementary Table 11a: Metabolite-metabolite correlations and correlations between metabolites and pro-inflammatory cytokines in control subjects.

Control		KYNA	TRP	QUIN	KYN	3HK	3HAA	XA	*IL1B*
TRP	r_s	**0.422***							
	Sig.	**0.011**							
QUIN	r_s	0.085	0.230						
	Sig.	0.628	0.184						
KYN	r_s	**0.926****	**0.398***	0.045					
	Sig.	**<0.001**	**0.018**	0.797					
3HK	r_s	**0.596****	**0.672****	0.320	**0.544****				
	Sig.	**<0.001**	**<0.001**	0.061	**<0.001**				
3HAA	r_s	**0.564****	**0.366***	0.131	**0.599****	**0.552****			
	Sig.	**<0.001**	**0.036**	0.469	**<0.001**	**<0.001**			
XA	r_s	**0.594****	**0.683****	-0.017	**0.496****	**0.806****	**0.483****		
	Sig.	**<0.001**	**<0.001**	0.925	**0.002**	**<0.001**	**0.004**		
IL1B	r_s	0.002	-0.175	-0.017	0.235	-0.075	0.282	-0.174	
	Sig.	0.992	0.356	0.930	0.212	0.695	0.146	0.358	
IL6	r_s	**0.576****	-0.032	0.118	**0.757****	0.210	0.358	0.027	**0.538****
	Sig.	**0.001**	0.871	0.543	**<0.001**	0.275	0.067	0.889	**0.003**

Abbreviations: KYNA: kynurenic acid; TRP: tryptophan; QUIN: quinolinic acid; KYN: kynurenine; 3HK: 3-hydroxykynurenine; 3HAA: 3-hydroxyanthranilic acid; XA: xanthurenic acid; IL1B: interleukin 1 beta; IL6: interleukin 6. *p<0.05, **p<0.01.

Supplementary Table 11b: Metabolite-metabolite correlations and correlations between metabolites and pro-inflammatory cytokines in major depressive disorder.

MDD		KYNA	TRP	QUIN	KYN	3HK	3HAA	XA	*IL1B*
TRP	r_s	**0.365***							
	Sig.	**0.015**							
QUIN	r_s	0.024	-0.206						
	Sig.	0.879	0.179						
KYN	r_s	**0.827****	0.178	0.259					
	Sig.	**<0.001**	0.252	0.093					
3HK	r_s	**0.689****	0.208	0.176	**0.530****				
	Sig.	**<0.001**	0.187	0.266	**<0.001**				
3HAA	r_s	**0.345***	0.032	0.139	**0.534****	0.117			
	Sig.	**0.040**	0.853	0.420	**<0.001**	0.503			
XA	r_s	**0.537****	**0.348***	0.001	0.261	**0.567****	-0.134		
	Sig.	**<0.001**	**0.022**	0.997	0.095	**<0.001**	0.435		
IL1B	r_s	0.086	-0.285	0.096	**0.382***	0.055	**0.388***	-0.006	
	Sig.	0.603	0.079	0.56	**0.018**	0.748	**0.028**	0.972	
IL6	r_s	0.001	-0.202	0.173	0.209	-0.005	0.33	0.154	**0.699****
	Sig.	0.993	0.225	0.300	0.215	0.977	0.07	0.363	**<0.001**

Abbreviations: KYNA: kynurenic acid; TRP: tryptophan; QUIN: quinolinic acid; KYN: kynurenine; 3HK: 3-hydroxykynurenine; 3HAA: 3-hydroxyanthranilic acid; XA: xanthurenic acid; IL1B: interleukin 1 beta; IL6: interleukin 6. *p<0.05, **p<0.01.

Supplementary Table 12a: Correlations between the KYNA/KYN ratio and the gene expression of KAT enzymes.

Diagnosis	Gene		KYNA/KYN
Control	*KYAT1*	r_s	-0.081
		p	0.670
	KYAT2	r_s	**0.382***
		p	**0.037**
MDD	*KYAT1*	r_s	**-0.393***
		p	**0.016**
	KYAT2	r_s	0.041
		p	0.807

*Correlation is significant at the 0.05 level.

Supplementary Table 12b: Correlations between the 3HK/KYN ratio and gene expression of the KMO enzyme.

Diagnosis	Gene		3HK/KYN
Control	*KMO*	r_s	0.274
		p	0.142
MDD	*KMO*	r_s	0.180
		p	0.285

Supplementary Table 12c: Correlations between the KYNA/KYN ratio and gene expression of the KAT enzymes across the suicide subgroups.

Suicide subgroup	Gene		KYNA/KYN
Control	*KYAT1*	r_s	-0.1091
		p	0.600
	KYAT2	r_s	**0.390***
		p	**0.037**
MDD-NS	*KYAT1*	r_s	**-0.509***
		p	**0.016**
	KYAT2	r_s	-0.281
		p	0.206
MDD-S	*KYAT1*	r_s	-0.067
		p	0.865
	KYAT2	r_s	**0.800****
		p	**0.010**

* Correlation is significant at the 0.05 level;
**Correlation is significant at the 0.01 level.

Supplementary Table 12d: Correlations between the 3HK/KYN ratio and gene expression of the KMO enzyme.

Suicide subgroup	Gene		3HK/KYN
Control	*KMO*	r_s	0.273
		p	0.144
MDD-NS	*KMO*	r_s	0.261
		p	0.218
MDD-S	*KMO*	r_s	-0.119
		p	0.779

* Correlation is significant at the 0.05 level; ** Correlation is significant at the 0.01 level.

Supplementary Table 13: Spearman's correlations between age and kynurenine pathway metabolites, genes and relevant glial/inflammatory markers.

Measure		Age Control	Age MDD
KYNA	r_s	0.262	0.222
	Sig.	0.140	0.152
TRP	r_s	-0.135	-0.070
	Sig.	0.439	0.652
QUIN	r_s	-0.078	**0.575****
	Sig.	0.664	**<0.001**
KYN	r_s	0.208	**0.313***
	Sig.	0.246	**0.044**
3HK	r_s	-0.186	0.134
	Sig.	0.284	0.398
3-HAA	r_s	-0.135	0.320
	Sig.	0.478	0.065
XA	r_s	-0.279	-0.142
	Sig.	0.122	0.362
KYAT1	r_s	**0.452***	**0.424****
	Sig.	**0.012**	**0.008**
KYAT2	r_s	0.041	-0.086
	Sig.	0.828	0.604
KMO	r_s	-0.224	-0.308
	Sig.	0.234	0.056
KYNU	r_s	**0.371***	**0.389***
	Sig.	**0.047**	**0.016**
HAAO	r_s	**0.367***	-0.077
	Sig.	**0.046**	0.646
QPRT	r_s	**0.482****	0.210
	Sig.	**0.007**	0.200
AIF1	r_s	0.313	0.198
	Sig.	0.093	0.228
GFAP	r_s	**0.542****	**0.525****
	Sig.	**0.002**	**<0.001**
IL1B	r_s	0.348	0.284
	Sig.	0.06	0.084
IL6	r_s	**0.385***	0.140
	Sig.	**0.048**	0.417
CX3CR1	r_s	0.087	0.063
	Sig.	0.647	0.704
AQP4	r_s	0.305	0.188
	Sig.	0.101	0.251

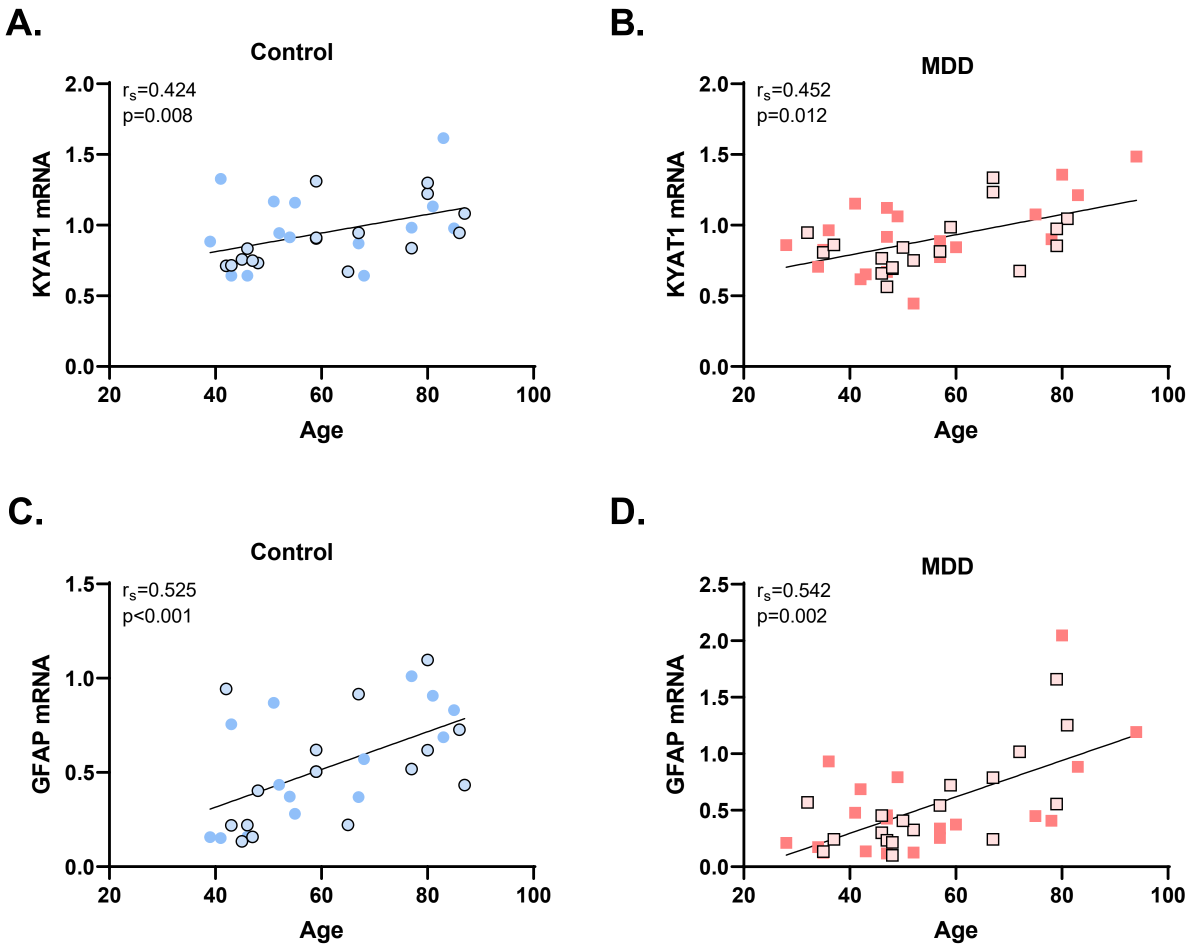

Supplementary Figure 8: (**A**) There was a significant positive correlation between *KYAT1* mRNA and age in controls (r_s=0.424, p=0.008) (**B**) and in MDD (r_s=0.452, p=0.012). (**C**) There was a significant positive correlation between *GFAP* mRNA and age in controls (r_s=0.525, p<0.001) (**D**) and in MDD (r_s=0.542, p=0.002). Males are represented by the outlined shapes.
